# Breaking free: endocytosis and endosomal escape of extracellular vesicles

**DOI:** 10.20517/evcna.2023.26

**Published:** 2023-06-30

**Authors:** Laís Ribovski, Bhagyashree Joshi, Jie Gao, Inge Zuhorn

**Affiliations:** ^1^Department of Biomedical Engineering, University Medical Center Groningen, University of Groningen, Groningen 9713 AV, the Netherlands.; ^2^Department of Bionanoscience, Kavli Institute of Nanoscience, Delft University of Technology, Delft 2629 HZ, the Netherlands.; ^#^Authors contributed equally.

**Keywords:** Extracellular vesicles, endocytosis, intracellular trafficking, functional delivery, endosomal escape

## Abstract

Extracellular vesicles (EVs) are natural micro-/nanoparticles that play an important role in intercellular communication. They are secreted by producer/donor cells and subsequent uptake by recipient/acceptor cells may result in phenotypic changes in these cells due to the delivery of cargo molecules, including lipids, RNA, and proteins. The process of endocytosis is widely described as the main mechanism responsible for cellular uptake of EVs, with endosomal escape of cargo molecules being a necessity for the functional delivery of EV cargo. Equivalent to synthetic micro-/nanoparticles, the properties of EVs, such as size and composition, together with environmental factors such as temperature, pH, and extracellular fluid composition, codetermine the interactions of EVs with cells, from binding to uptake, intracellular trafficking, and cargo release. Innovative assays for detection and quantification of the different steps in the EV formation and EV-mediated cargo delivery process have provided valuable insight into the biogenesis and cellular processing of EVs and their cargo, revealing the occurrence of EV recycling and degradation, next to functional cargo delivery, with the back fusion of the EV with the endosomal membrane standing out as a common cargo release pathway. In view of the significant potential for developing EVs as drug delivery systems, this review discusses the interaction of EVs with biological membranes en route to cargo delivery, highlighting the reported techniques for studying EV internalization and intracellular trafficking, EV-membrane fusion, endosomal permeabilization, and cargo delivery, including functional delivery of RNA cargo.

## INTRODUCTION

Extracellular vesicles (EVs) are lipid bilayer vesicles that are secreted by cells and contain a diverse array of cargo, including proteins, lipids, DNA, and RNA. They play a role in cell-cell communication through the delivery of their cargo to recipient cells and thereby influencing cellular processes such as proliferation, differentiation, and immune responses^[1-3]^. EVs can be divided into three subpopulations according to their biogenesis route and size: exosomes are formed through the inward budding of the late endosome and have a size between 30-150 nm, microvesicles or ectosomes (150-2,000 nm) are created through outward budding of the plasma membrane, and apoptotic bodies (500-4,000 nm) are the result of outward blebbing of the plasma membrane during apoptosis. The different populations of vesicles are highly heterogeneous, and their methods of isolation and classification are under constant scrutiny. The MISEV 2018 guidelines suggested EV-enriched markers to identify the subcellular origin of secreted vesicles and if those could not be validated to refer to small EVs (sEVs) or large EVs (LEVs) purely on the basis of their size, i.e., being smaller or larger than 200 nm, respectively^[4]^. Thanks to the development of innovative methods to study EV biogenesis, endocytosis and cargo release, researchers have started to unveil EV structure-function relationships, leading to a vast increase in knowledge on EV behavior and function.

With regard to the formation of EVs, a few main regulators and machineries can be pointed out. Exosomes originate from late endosomes upon the inward budding of the endosomal membrane, forming intraluminal vesicles (ILVs). When the multivesicular body (MVB) fuses with the plasma membrane, the ILVs are released into the extracellular space and are now called exosomes^[5,6]^. Besides small GTPase activity, both endosomal sorting complex required for transport (ESCRT)-dependent and ESCRT-independent mechanisms control the formation and release of ILVs^[7-9]^. Microvesicles are plasma-derived vesicles originating from the outward budding of the plasma membrane. The vesicles are released directly after formation and fission, which involves the ESCRT machinery together with small GTPases^[10,11]^. Apoptotic bodies are a product of programmed cell death, i.e., apoptosis. Cells start to bleb and disassemble into membrane-enclosed fragments, named apoptotic bodies, which are typically heterogeneous in size and content^[12]^.

Size and composition are highly heterogenous both between and within EV populations. Besides size and surface composition heterogeneity, cargo heterogeneity is also observed among subpopulations of EVs, e.g., in exosomes^[13,14]^. Differential centrifugation and size exclusion chromatography are two largely employed methods to isolate EVs and separate subpopulations based on size. Immunoaffinity-based methods are effective options for highly selective populations, but are not suitable for large-scale production and limited to the use of validated marker proteins for which antibodies exist with high affinity and specificity. Polymer-based precipitation of EVs is simple and provides a high yield, but at the cost of heterogenous populations^[15-17]^. Combinations of the above-mentioned methods may be beneficial for isolating specific populations or enhancing purity and yield^[18-21]^.

Because the content of EVs reflects the physiological state of the cells they are derived from, understanding the fundamental biological processes that control extracellular vesicle biogenesis and trafficking holds great potential to develop EVs as drug delivery systems or signaling pathway modulators within disease contexts. This review discusses the current knowledge on cellular interactions with EVs, their uptake and intracellular trafficking, and cargo delivery, highlighting the reported techniques for studying endocytosis and cargo release.

## EV INTERNALIZATION AND INTRACELLULAR TRAFFICKING - BREAKING THROUGH MEMBRANE BARRIERS

### EV-cell membrane interactions

The mechanism of internalization of EVs by recipient cells is the first essential step that defines their intracellular route, intracellular fate and functionality. Besides fusion with the plasma membrane, endocytosis is most often described as the mechanism of uptake of EVs^[22,23]^, including clathrin-mediated endocytosis, phagocytosis, macropinocytosis and lipid raft-mediated endocytosis, including caveolae-mediated endocytosis^[6,24-33]^. Interestingly, molecular determinants of other lipid raft-dependent endocytic pathways, specifically flotillin, ARF6 and RhoA, were reported to play a role in EV biogenesis/release^[11,34,35]^. Similar routes of internalization have been observed for a.o. viruses and synthetic nanoparticles and are known to be cell type-dependent as well as dependent on nanoparticle characteristics^[36-39]^.

#### EV binding to recipient cells

How EVs interact with the cell surface and the route that EVs follow after subsequent internalization will depend on properties and conditions such as size, composition, cell source, ligands, environmental pH, presence of serum, and even isolation method^[1,20,40-42]^ A size-dependent internalization of nanoparticles via distinct endocytic routes was shown for nanoparticles with fixed sizes^[43]^. Obviously, because of the heterogeneity in the size of EV populations, such a relationship is difficult to detect for EVs, further complicated by a probable heterogeneity in (surface) composition of EVs of different sizes. Using targeted gold nanoparticles of different sizes, it was shown that small particles (15-30 nm) have a higher cell binding probability than larger particles (90-150 nm), but result in a lower amount of mass bound per cell^[44]^. However, also here caution is needed, as the ligand density on the gold nanoparticles of different sizes may differ. Overall, varying one parameter, e.g., size, while keeping all other parameters, including ligand density, constant is a major challenge, also for synthetic nanoparticles.

The binding of EVs to recipient cells is a prerequisite for EV endocytosis. One class of proteins that facilitates this interaction is tetraspanins, a class of membrane-spanning proteins. Tetraspanins are commonly found in EVs and play a role in EV biogenesis, cargo sorting, cellular uptake, and functionality. While tetraspanins are typically enriched within exosomes, other subpopulations of EVs may also contain tetraspanins. CD9, CD63, and CD81 are examples of tetraspanins that are associated with EV-cell interaction^[45,46]^. For example, researchers have shown that blocking CD9 can reduce the uptake of cancer cell fibroblast (CAF)-derived EVs by pancreatic cancer cells, cancer cell migration, and epithelial-to-mesenchymal transition (EMT). This finding reveals an important role for EVs in the interaction of the tumor with the tumor microenvironment and its aggressiveness^[1]^.

In contrast, a low CD9 expression on sEVs produced by colorectal cancer cells was shown to promote their uptake^[47]^, which could be explained by the inactivating effect of CD9 on ADAM17-mediated adhesion of sEVs with α5β1 integrin on recipient cells. Tetraspanins are known to interact with lipids and other integral membrane proteins, including integrins, forming so-called tetraspanin-enriched microdomains (TEMs). TEMs play a role in the regulation of adhesion strength, and viruses have been shown to enter cells through TEMs, with or without direct binding to tetraspanins^[48]^. Interestingly, CD9 can dictate the mechanism of coronavirus (CoV) entry. Specifically, CoV particles entered via fusion with the plasma membrane in the presence of CD9, whereas in its absence, viral particles were taken up via endocytosis, followed by fusion with the endosomal membrane^[49]^. In a recent paper, CD63 and CD9 knockdown in producer cells and recipient cells was used to indicate that these tetraspanins were not required for EV uptake and content delivery^[50]^. Unfortunately, the mechanism of uptake, i.e., whether this occurred via plasma membrane fusion or endocytosis, was not evaluated. Moreover, downregulation of the expression of a specific protein may result in compensatory expression of other proteins, which may take over (part of) its function.

Besides tetraspanins, also integrins are membrane-bound proteins found in EVs that mediate their interaction with cells. Integrins are adhesion molecules that mediate cell-cell adhesion and cell adhesion to the extracellular matrix^[51]^. Likewise, in EVs, the integrins support adhesion to the extracellular matrix and recipient cells, providing the means for targeting. Vice versa, inhibition of integrin activity can be used to reduce the uptake of EVs by recipient cells^[52]^. For example, disintegrin inhibitor (DisBa-01) that inhibits αvß3 integrins, YIGSR peptide that inhibits β1-containing integrins, and RGD peptides that inhibit RGD-binding integrins such as αvβ3, αvβ5, αvβ6, αvβ1, αvβ8, α5β1, αIIbβ3, and α8β1, have been shown to reduce the uptake of EVs by specific cell types^[53-56]^.

Thirdly, the glycan composition of EVs mediates cell interaction and may confer cell specificity. sEVs isolated from adipose-derived stem cells (ADSC-sEVs) were deglycosylated by means of α2-3,6,8 neuraminidase, ß1-4 galactosidase, ß-N-acetylglucosaminidase or a combination of those enzymes^[42]^. Cellular uptake of the ADSC-EVs by HeLa cancer cells was reduced for deglycosylated EVs, but when the EVs were exposed to non-malignant human lung fibroblasts, deglycosylated EVs showed higher uptake than the untreated control. Glycan engineering of EVs with sialyl Lewis X and Lewis X ligands increased cellular specificity for endothelial and dendritic cells, respectively^[57]^. These results reinforce that EV glycan profile, including glycoproteins and glycolipids, has an impact on the interaction of EVs with cells.

**Figure 1:**
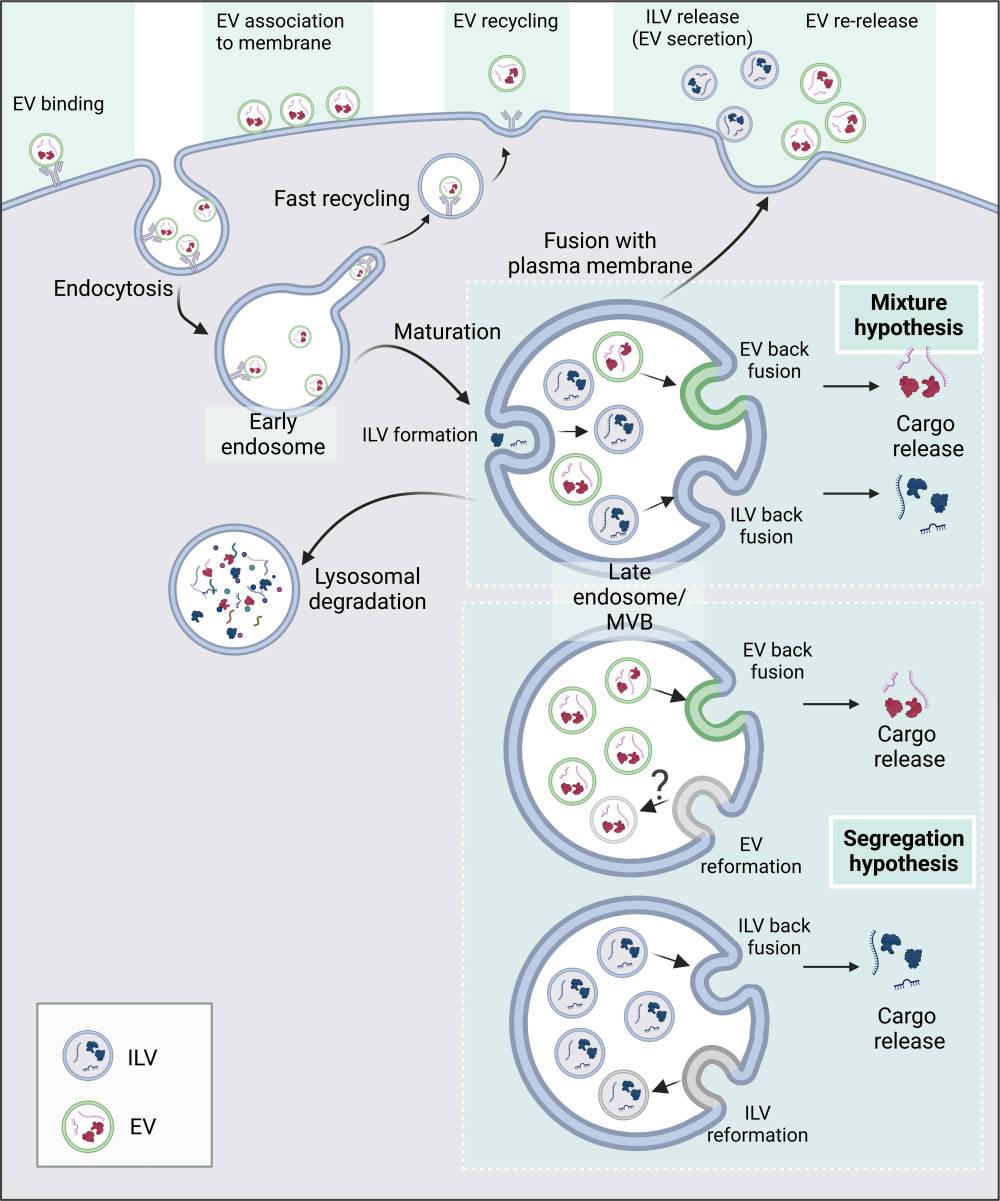
EV biogenesis and cellular processing. EV endocytosis, intracellular trafficking, and cargo release may result in phenotypic changes in recipient cells. It remains to be established if, within MVBs, there is a mixed presence of newly-formed ILVs and internalized EVs (mixture hypothesis) or that MVBs contain either ILVs or EVs (segregation hypothesis). This image was created with BioRender (https://biorender.com/).

It is important to notice that an increase in the intracellular accumulation of EVs is generally referred to as an increase in "uptake". However, enhanced accumulation of EVs does not necessarily mean that there was an increase in internalization of EVs. Equally possible is their entrapment within the cell due to enhanced evasion of degradative and/or recycling pathways [Figure 1].

#### Environmental factors influencing the cellular secretion and uptake of EVs

Environmental factors can affect EV biogenesis, release, and cellular uptake. Cancer cells are shown to secrete large amounts of EVs that reflect characteristic stress-related phenotypic changes in tumor cells induced by, e.g., acidic pH and hypoxia, i.e., hallmarks of the tumor microenvironment (TME). Interestingly, the number of secreted EVs at acidic pH was shown to be reduced even though the protein concentration was increased compared to pH 7.4 conditions^[41,58]^. Therefore, when determining EV amounts, it is important to report not only EV protein content, but also EV numbers. Environmental pH influences not only the production of EVs, but also EV uptake. Intriguingly, EVs secreted at low pH (pH 5) were more efficiently internalized than EVs secreted at pH 7 (both at environmental pH 5 and 7) when in the absence of serum, but the opposite behavior occurred in the presence of serum^[41]^. Overall, spontaneous uptake of EVs occurs at a low rate of about ~1% at 1 h^[59]^. However, EVs intrinsic targeting to specific cell types offers an advantage that can be exploited for the delivery of therapeutics^[60]^. Natural or engineered surface ligands are crucial determinants of the uptake and uptake rate of EVs. Under hypoxic conditions, endothelial cells exhibited a higher uptake of EVs compared to normoxic conditions. Additionally, changes were observed in both the quantity and composition of EVs that were secreted under hypoxic conditions^[61-64]^. Interestingly, Cerezo-Magaña *et al.* observed a correlation between hypoxia-stimulated EV uptake and increased heparan sulfate proteoglycan (HSPG) endocytosis via lipid raft-mediated endocytosis^[64]^. The examples given above illustrate the interplay between environmental factors (e.g., presence of serum, pH, hypoxia), EV release and EV uptake.

In addition, non-physiological stimuli can be used to enhance EV secretion. Low electricity levels applied to murine melanoma B16F1 and murine fibroblast 3T3 Swiss Albino cells increased EV secretion, while it did not alter the uptake of these EVs exposed to the same cell types in the absence of electrical stimulation^[65]^. We should mention that the authors did not evaluate EV uptake by cells that were stimulated by electricity; thus, the relation between electrical stimulation of cells and EV uptake remains to be investigated. Importantly, EV size distribution and zeta potential were unaffected by electrical stimulation of the producer cells, as was the expression level of CD9, HSP70 and CD81, which may explain the unaltered uptake of the EVs. The production of EVs is a process of relatively low yield and efforts to increase the yield are fundamental to translating EVs to the clinic, e.g., through the use of bioreactors and stimuli that boost release while maintaining consistent production of EVs with defined content^[66]^.

Employing high-speed atomic force microscopy (HS-AFM), Sajidah *et al.* observed nanotopological changes in sEVs caused by stress conditions^[67]^. Low (4.0) and high (10.0) pH were compared to pH 7.5 (near physiological conditions), temperature was changed from 4 to 100 °C and compared to 37 °C, and physiological buffer condition (50 mM Tris HCl with 150 mM NaCl) was compared to hypotonic and hypertonic environments, i.e., 0.0 M NaCl and 1.8 M NaCl, respectively. Low pH did not affect the sEVs morphology, which may have been expected because EVs originate from acidic MVBs. Hypotonic conditions also showed preservation of the spherical morphology of the sEVs, whereas high temperature (60 and 100 °C), high pH (pH 10) and hypertonic conditions (1.8 M NaCl) were damaging to the structure of the EVs. This may set limitations to EV isolation and sterilization procedures.

#### Intracellular trafficking of EVs

The mode of internalization of EVs has a great impact on their subsequent intracellular trafficking and fate. EVs that are internalized via endocytosis may follow the canonical pathway through early endosomes, late endosomes to (degradative) lysosomes. Phenotypic changes in recipient cells can be triggered through the activation of cell signaling pathways upon binding of EVs to cells and/or the release of functional cargo into the cell cytosol. Membrane fusion enables cargo release from EVs into the cytosol. This fusion process can occur at the plasma membrane level^[58]^ and the endosome level, the latter being known as back fusion^[22,59,68-70]^. Membrane fusion can be stimulated by the presence of fusogenic proteins in EVs. Syncytin-1, syncytin-2, VSV-G and Hur proteins are known for their fusogenic activity^[71-73]^. Overexpression of Syn 1 in EVs was shown to boost the uptake and cargo delivery of EVs in acceptor cells^[72]^. Even at endogenous levels of Syn 1, Uygur *et al.* showed that GFP gene transfer occurred between GFP-transduced and non-transduced cells by means of Syn 1-mediated fusion of extracellular membrane vesicles (EMVs, i.e., EVs plus retroviral particles) with cells^[74]^. In contrast, Somiya *et al* reported that EV-mediated cargo delivery did not occur unless the EVs were engineered to express the VSV-G fusion protein^[75,76]^. However, the EVs were isolated by PEG precipitation and the presence of residual PEG in the EV formulation could have influenced the fusion capacity of the EVs^[77]^. When Zhang and Schekman investigated the intercellular transfer mechanism of Cas9 protein and split GFP fragments, they showed that direct cell-cell contact was a requirement for effective protein transfer between donor and acceptor cells. Specifically, syncytin-2-dependent membrane fusion at the contact point between a microtube (2-4 µm diameter) on one cell and the plasma membrane of another cell was necessary for the formation of an open-ended tubular connection between the cells^[73]^. Conversely, accumulating evidence supports protein-independent fusion of EVs with biological membranes.

It has been suggested that proteins play a minor role in the fusion process, as paraformaldehyde treatment of EVs (crosslinking of EV proteins) did not alter their fusogenic properties^[22,78]^. Still, membrane proteins seem to be needed as structural elements contributing to membrane fusion, because lipid vesicles mimicking cell membrane lipid composition or EVs solubilized with octylglucoside (removing proteins from the membrane) abrogated fusion activities^[58,79]^. In addition, EV fusion increased for EVs that were generated by cells grown under acidic conditions, which correlated with a decrease in membrane fluidity due to a change in lipid composition. Specifically, an increase in sphingomyelin (SM), cholesterol, and GM3 was detected and suggested to promote fusion^[58,80,81]^.

By showing the absence of endosomal permeabilization, using a galectin-3 assay (see *Techniques to study endosomal permeabilization*), and detecting cargo exposure to the cytosol, using GFP-loaded EVs and GFP fluobody-expressing recipient cells (see *Techniques to study EV-membrane fusion and cargo delivery*), Joshi *et al.* showed that EVs are taken up via endocytosis and a fraction of them (~25%) release their cargo in a non-disruptive manner. Moreover, the site of cargo release was identified through correlative light and electron microscopy (CLEM), revealing EV back fusion with endosomes^[22]^.

Bonsergent *et al.* corroborated these findings, showing a very similar efficiency of the cargo release process (30%), but using a different analytical method (NanoLuc; see *Read out systems for functional delivery of RNA cargo*)^[59,82]^. Cells incubated with EVs loaded with NLuc-HSP70 showed only ~1% uptake in recipient cells and the uptake never reached saturation even at very high concentrations (100 ug/ml), leading the authors to suggest that EVs are endocytosed nonspecifically, i.e., without associating to a specific receptor, in line with a previous study^[70]^. Using cell fractionation, they showed that roughly 20%-30% of the internalized EV cargo was present free in the cytosol of the cells. This release was not because of endosomal permeabilization as EVs did not induce galectin-3 punctae, again corroborating results from Joshi *et al*^[22]^. Interferon-inducible transmembrane (IFITM) proteins IFITM 1 and 3 showed 80%-90% colocalization with internalized EVs when overexpressed in HEK293T cells^[83,84]^, and their presence inhibited the cargo release from EVs. IFITM proteins are known to inhibit fusion reactions during viral infection^[83,85,86]^, and may inhibit EV membrane fusion through similar mechanisms. Overall, the rate-limiting step in EV cargo delivery appears to be the EV uptake (~1%), while the content delivery is much more efficient.

When studying EV cargo release upon interaction with plasma membrane sheets, efficient cargo release was observed, which was pH-dependent and proteinase K-sensitive^[82]^. Using another cell-free assay, Morandi *et al.* provided interesting insights into the process of endosomal fusion in EV cargo release^[22,68,87]^. Using a fluorescence resonance energy transfer (FRET) assay (see *Techniques to study EV-membrane fusion and cargo delivery*), EVs were shown to fuse with large unilamellar vesicles (LUVs) mimicking late endosomal composition, i.e., without cholesterol and with LBPA, a lipid with pH-dependent fusogenic properties. Fusion only happened at acidic pH and was significantly reduced when the EVs were treated with proteinase K, suggesting that EV membrane proteins are required in this fusion process, in line with previous reports^[59,82]^. It is important to note that the recipient LUVs did not contain proteins, meaning that the proteins in the endosomal membranes may be dispensable for fusion and the proteins in EVs may play a more structural than functional role, which was suggested before^[58]^. EVs did not fuse with LUVs with early endosomal membrane composition, underscoring the importance of specific lipids in the fusion process^[22,87]^.

Nevertheless, there is speculation of a putative EV fusogen or set of fusogens akin to viral fusion proteins that drive the process of membrane fusion^[88]^. Most viral fusogens require activation by low pH, and similarly, EV fusogens may get activated by acidic pH. Further, the EV membrane fusion process follows the canonical intermediates of viral fusion, namely (A) close contact between lipid bilayers; (B) formation of a hemifusion diaphragm by fusion with the outer leaflet; (C) initial fusion pore formation by fusion of the inner leaflet; (D) expansion of the fusion pore resulting in content mixing^[89-92]^. All in all, EV fusion features that play a role in cargo release are strikingly similar to that of some viruses. And both EVs and viruses hijack the host cell machinery for the expression of their genetic material. That putative EV fusogens may exhibit structural and molecular similarities to viral fusogens has already been suggested in many studies^[22,26,59,69]^.

However, as EVs (ILVs) are generated in an acidic milieu already, there must be a mechanism to activate the EV fusogen after the EVs are released and taken back into cells. Intriguingly, when EVs are treated with acidic pH, neutralized with buffer, and allowed to fuse with endosomes at acidic pH, they readily fuse with the endosomal membranes with comparable efficiency as non-treated EVs. This suggests that these putative EV fusogens indeed can get reversibly activated^[22,87]^. Thus, EVs present a paradoxical scenario: ILVs are formed in an acidic environment, i.e., in MVBs, without undergoing massive back-fusion, whereas following the endocytosis of EVs, cargo release by means of back-fusion occurs upon endosomal acidification. This raises the question of how fusion activation at low pH is compatible with ILV biogenesis in MVBs^[68,93,94]^. Moreover, the occurrence of back fusion of ILVs has been reported^[87]^, suggesting that a general block of ILV back fusion does not exist. We hypothesize that the fusion propensity of ILVs/EVs with endosomal membranes is controlled by physicochemical properties, e.g., macromolecular crowding or liquid-liquid phase separation^[94-97]^. Alternatively, the non-fusogenic ILVs in MVBs may represent the population of ILVs that was generated in early endosomes and lacks LBPA. Similarly, the absence of LBPA in early endosomes^[98]^ was held responsible for the absence of release of genetic cargo from early endosomes, as observed in non-viral gene delivery using antisense oligonucleotides and polyplexes^[99,100]^.

Likely, fusogenicity is context-dependent and will vary among and within EV subpopulations, while being affected by the source cells and their state. This may partially explain the differential impacts of EVs from different sources under different conditions. For example, there are differences in EV behavior between different donor-recipient cell combinations, underscoring the possibility that different cells may use different pathways for EV endocytosis and may differently regulate EV cargo release.

Next to inducing escape from endosomes, EVs can avoid lysosomal degradation through recycling pathways. Rab11 is a marker of recycling endosomes and has been implicated in the tethering and homotypic fusion of vesicles^[101,102]^. Together with other Rab GTPases, e.g., Rab27 and Rab35, Rab11 promotes EV sorting and recycling^[103,104]^.

### Assays for detection and quantification of EV internalization and intracellular trafficking

Analytical methods to determine the internalization mechanism of EVs, their subsequent intracellular trafficking, as well as cargo release are essential to further improve our understanding of how EVs interact with *in vitro* and *in vivo* systems [Table 1]. Fluorescently labeled EVs can be directly visualized in cells, while enzyme labeling allows for their detection through substrate conversion.

**Table 1:** Assays to study uptake mechanisms, intracellular localization, and functional cargo delivery by EVs

**EVs**	**Producer cells or source material**	**Recipient cells**	**Assay type**	**Uptake mechanism**	**Reference**
EV-GLA	Stable clones of CHO DG44 expressing GLA and HEK293T	Primary cultures of mouse aortic endothelial cells (MAEC) derived from Fabry KO mice and HEK293 cells	Pharmacological inhibitors of endocytosis; DiOC, DiD, or DiR labeled EVs	Clathrin-mediated endocytosis and macropinocytosis	^[24]^
placenta-derived sEVs	Primary human trophoblasts, Stable clones BeWo cells transfected with mCherry-TSG101-NanoLuc	Primary placental fibroblasts (PPF) and primary human uterine microvascular endothelial cells (HUtMEC)	Pharmacological inhibitors of endocytosis; siRNA-mediated knockdown of specific pathways; NanoLuc activity; DiI-labeled EVs loaded with NanoLuc protein; Alexa488-labelled microRNA-517a	Clathrin-mediated endocytosis and macropinocytosis	^[25]^
DSP1-Tetraspanin EVs	Stable clones of SUM159 cells transfected with DSP2 and DSP1-CD9	DSP2-expressing SUM159 cells and SUM159 cells	Pharmacological inhibitors of endocytosis; DSP reporter proteins (EGFP and luciferase); Colocalization with EEA1 and CD63	Dynamin2-dependent/Clathrin-mediated endocytosis	^[70]^
grapefruit-derived EVs	Grapefruit juice	HaCaT cells	Pharmacological inhibitors of endocytosis; DiO-labeled EVs; TAMRA-labeled siRNA; siRNA-mediated gene knockdown	Clathrin-mediated endocytosis	^[113]^
NLuc-Hsp70-EVs, NLuc-CD63-EVs, GFP-Hsp70 EVs	Stable clone of HeLa cells transfected with NLuc-Hsp70, Nluc-HsP70 or GFP-Hsp70 and of HEK293T transfected with flag-tagged IFITM1 and IFITM3	HeLa cells, HEK293T cells, HEK293T overexpressing Flag-tagged IFITM1 or 3	Colocalization with Rab5 and Lamp1; Cytosolic and membrane fraction separation + NLuc activity; Pharmacological inhibitor (BafA1); Inhibition by IFITM proteins (fusion)	Membrane fusion	^[59]^
Syn1-EVs,VSV-G EVs carrying FRB-NLuc-HA or NLuc-Hsp70	Stable clone HeLa cells transfected with NLuc-Hsp70 or CD8-GFP, HT1080 and HeLa cells overexpressing Syn1 and VSV-G	HeLa cells, GFP-PEST HT1080 acceptor cells	NLuc activity; Syncytia formation	Membrane fusion	^[72]^
Normoxic U87 and GL261 EVs	Normoxic U87 MG or GL261 cells	U87 MG and GL261 cells, and primary patient-derived human GBM cell lines U3034, U3065, and U3082 (HGG cells)	Colocalization with transferrin, dextran, cholera toxin B (raft marker); PKH67 green or PKH26 red-labeled EVs	Raft–mediated endocytosis (heparan sulfate proteoglycan endocytosis)	^[64]^

GLA: alpha-galactosidase A; Syn1: Syncytin-1; BafA1: bafilomycin A1; Nluc: NanoLuc luciferase; DSP: Dual Split; NanoLuc luciferase-tagged Hsp70: NLuc-Hsp70; FRB: FKBP-rapamycin binding; HGG: high-grade glioma

Detection of EVs can be obtained by labeling the EVs employing fluorescent dyes such as DiI, DiO, PHK26 and PHK76 membrane dyes, and CFDA and calcein AM membrane-permeable dyes^[25,56,61,105-107]^. Although easy to use, labeling of EVs with fluorescent dyes has its downsides. EV membrane labeling with lipophilic dyes may change EV characteristics, influencing their behavior, and may suffer from exchanges with other biological membranes, causing faulty identification of EV localization in cells^[108]^. To ensure that the fluorescence signal accurately reflects the localization of EVs, a non-exchangeable dye should be used and free dye should be used as a control in the experiments. Finally, it should be verified if labeled and unlabeled EVs show the same functional effect in recipient cells to exclude a possible effect of EV labeling on EV function.

Alternatively, reporter proteins, for example, GFP, YFP, mCherry fluorescent protein, or nanoluciferase and *Gaussia* luciferase bioluminescent proteins, are used for EV labeling^[22,25,59,72]^. Fluorescence or bioluminescence detection using microscopy, flow cytometry and spectroscopy are then employed to determine EV uptake by cells, although early stages in EV uptake, i.e., binding versus internalization, are difficult to discern due to the resolution limit of conventional light microscopes. Toribio *et al.* described a luciferase/GFP-based assay that is sensitive enough to quantify and trace EV uptake at early stages employing a pair of chimeric reporter proteins, Dual Split 1 (DSP1) and Dual Split 2 (DSP2)^[70]^. DSP1 was fused to the N-terminus of CD9 and co-expressed with DSP2 in EV producer cells. Recipient cells were preloaded with luciferase substrate and incubated with DSP1-CD9/DSP2 EVs, i.e., carrying luciferase activity. Upon their internalization, EVs get exposed to the luciferase substrate, which will be converted, resulting in a luminescent signal. The subcellular localization of EVs was detected by the GFP fluorescence signal. Finally, labeling the content of the EVs, e.g., mRNA, is also an option^[25]^.

Inhibition or competition assays can be employed to characterize specific uptake routes or (receptor-mediated) endocytosis pathways [Table 1]. Examples of classical pharmacological inhibitors of endocytosis are dynasore (dynamin-dependent endocytosis), amiloride (macropinocytosis); genistein (caveolae-dependent/clathrin-independent endocytosis); chlorpromazine and Pitstop 2 (clathrin-mediated endocytosis); cytochalasin D (actin-dependent endocytosis) and bafilomycin A1 (endosomal acidification)^[24,25,39,59]^. Importantly, pharmacological inhibitors of endocytosis are non-specific and warrant the use of alternative approaches to study nanoparticle/EV endocytosis. Moreover, investigation of the mechanisms of nanoparticle internalization has revealed that inhibition of specific endocytic pathways may result in the upregulation of other pathways, and that not all uptake pathways result in functional cargo delivery^[100,109-112]^. Therefore, adding a functional experiment to confirm that a proposed mechanism is involved in enhancing or abrogating the functional outcome is highly recommended.

The cellular uptake of EVs can be deduced from observing a functional effect, i.e., a specific response of cells upon their exposure to EVs, which is associated with the EVs content. For example, EVs loaded with siRNA may trigger the degradation of a specific mRNA which can be examined by quantifying the mRNA or protein level^[113]^. Recipient cells that are transfected with reporter genes coding for fluorescent or bioluminescent proteins can be used to quantify EV-mediated siRNA silencing efficacy by means of fluorescence detection.

## INTRACELLULAR DELIVERY OF EV CARGO

Functional delivery by EVs is determined by their ability to release the cargo contained in them into the recipient cell. Various aspects need to come together to achieve functional EV-mediated delivery: cellular uptake; intracellular trafficking; cargo release; functional outcome. While the uptake of EVs is heavily researched, we are just beginning to understand the molecular basis of intracellular trafficking and cargo release by EVs.

After uptake, EVs generally end up in endosomes^[22,64]^, although uptake via plasma membrane fusion^[58]^ is also a possibility, which will result in immediate release of EV content into the cell cytosol. Early endosomes are known to mature into late endosomes that ultimately fuse with lysosomes, although endosomal sorting can also result in recycling of endocytic cargo. Such recycling or re-release has been described for EVs^[114]^. The induction of phenotypic changes in recipient cells by EVs provides indirect evidence for the functional delivery of their cargo, evading degradation within lysosomes. Multiple studies have shown that EVs are able to escape endosomes by membrane fusion with the endosomes and this seems to be the primary pathway of EV cargo release^[68,75,76]^. Contrastingly, some studies point to a lack of cargo release due to the failure of EVs to undergo endosomal escape in their native form, which can be overcome through EV surface functionalization with a fusogen such as VSV-G^[75,76]^. In addition, alternate pathways have been suggested, including cargo release through endosomal permeabilization and nuclear import of EV cargo from Rab7+ endosomes through nuclear pores^[46,115]^. Taken together, EV uptake, recycling, and degradation determine the extent of intracellular EV accumulation, while EV fusion with the plasma membrane or endosomal membrane, or endosomal rupture determines functional cargo delivery into the cell cytosol, of which back fusion with the endosomal membrane has been most widely described [Figure 1].

In the following section, we have listed the hitherto reported techniques to shed light on EV cargo release pathways. Thereafter, we discuss in detail various factors impacting the cargo delivery process. As the pool of methods for cargo delivery identification is still growing, we also lay out controls to be used in cargo delivery studies for reducing false positives and attaining valid conclusions.

### Techniques to study EV-membrane fusion and cargo delivery

#### Lipid mixing-based assays

The introduction of fluorescent probes, e.g., octadecyl Rhodamine B chloride (R18), in (artificial) membranes at self-quenching concentrations can be used to measure lipid mixing/membrane fusion^[116]^. Costrafeda *et al.* used liposomes containing equal amounts of phosphatidylserine (PS), phosphatidylcholine (PC), and cholesterol labeled with R18 (Chol-R18) to interact with EVs^[117]^. Chol-R18 dequenching upon its dilution as caused by the fusion of the PS:PC:Chol-R18 liposomes with EV membranes was visible as an increase in fluorescence. Similarly, Parolini *et al.* incubated cells with R18-labeled EVs to monitor an increase in fluorescence intensity as a measure for the fusion of the EVs with cellular membranes^[58]^.

Morandi *et al.* used a FRET-based lipid mixing assay together with cryogenic transmission electron microscopy (cryo-TEM) and electron cryotomography (cryo-ET) to provide important insights into the membrane fusion process between EVs and artificial membranes^[68]^. They probed the interaction of EVs that were labeled with the FRET pair DiI and DiD, with unlabeled liposomes. In intact EVs, DiI excitation results in DiD fluorescence emission, because of the close apposition of both fluorophores and the overlap between DiI emission wavelength and DiD excitation wavelength. In the case of fusion between EVs and liposomes, the distance between the FRET pair increases, resulting in an increase in the donor fluorescence intensity (and a concomitant decrease in acceptor fluorescence intensity). Cryo-TEM and cryo-ET revealed EV hemifusion intermediates and internal content mixing, respectively, which correlated with the membrane mixing data obtained with FRET.

However, the use of liposomes as cell mimics precludes the identification of the cellular location of cargo release, and in case cells are used, live cell imaging is required to visualize the site of membrane fusion, because of the temporary nature of a change in fluorescence intensity.

#### Nanobody-based assay

A pioneering technique to quantify EV-mediated cargo delivery that also pinpointed the subcellular site of EV cargo release was introduced by Joshi *et al.*^[22]^. Their assay used a combination of GFP-loaded EVs, mCherry-labeled anti-GFP nanobody-expressing recipient cells, and correlative light and electron microscopy (CLEM). Specifically, GFP was loaded inside EVs through the expression of GFP-CD63 in EV producer cells. The tagging of GFP to CD63 made sure that the GFP signal would not be diluted in the cytosol upon EV back fusion with the endosomal membrane. Further, recipient cells were genetically engineered to express anti-GFP fluobody in the cytosol. Upon incubation of fluobody-expressing cells with GFP-CD63 EVs, back fusion of EVs with the endosome led to GFP exposure to the cytosol, resulting in anti-GFP fluobody binding at the endosomal membrane and formation of fluobody punctae. This assay allowed for quantification of the number of EV-containing endosomes (GFP-positive) as well as the number of endosomes exposing EV cargo to the cell cytosol (GFP-mCherry double positive). Subsequent investigation of the fluobody punctae using CLEM revealed endosomes as the underlying cellular structures, providing direct evidence for EV cargo release from endosomes. Cargo release from endosomal structures was confirmed in another study by following quantum dot-labeled EVs carrying fluorescent miRNA in recipient cells containing fluorescently labeled endosomes through single particle tracking^[118]^.

#### Protease-based assays

Albanese *et al.* developed an assay to determine EV cargo delivery based on β-lactamase-mediated cleavage of the FRET substrate CCF4^[119]^. EVs were generated by cells expressing CD63- β -lactamase and incubated with recipient cells that were loaded with CCF4. Cleavage of CCF4 by β-lactamase was detected by a shift in fluorescence emission from 520 nm to 447 nm, indicating the exposure of β-lactamase from EVs to the cell cytosol. However, EV cargo release was not detected unless the EVs were surface functionalized with VSV-G through co-expression of CD63- β-lactamase and VSV-G in EV producer cells.

A couple of reports^[59,114]^ have shown the use of luciferase enzymes for the detection of EV cargo delivery, especially NanoLuc^[120]^, which gives a very high signal-to-noise ratio. The general idea is that a NanoLuc protein is loaded in EVs by overexpression in donor cells, and its delivery in recipient cells is quantified as luciferase activity upon the addition of a luciferase substrate. It can also be used in a converse strategy where recipient cells express luciferase and functional delivery of anti-luciferase miRNAs via EVs can be detected from a reduction in luciferase activity^[119]^.

Somiya *et al.* developed the EV-mediated tetraspanin-tetracycline transactivator (tTA) delivery (ETTD) assay^[75,76,121]^, in which a tetracycline-controlled transcription factor is delivered by EVs, which upregulates the expression of a TRE (Tetracycline responsive element)-controlled reporter gene (luciferase or fluorescence protein) in the recipient cells. Specifically, the tetraspanins CD9, CD63, or CD81 were fused at the C-terminal with the TEVp cleavage site, followed by tetracycline transactivator (tTA). When the EV membrane fuses with the endosome, the lumenal tTA is exposed to the cytosol, cleaved in the presence of TEVp expressed in the cytosol, and releases the transcription activator tTA. The cytoplasmic release of tTA leads to the tTA induction of the reporter gene expression under the TRE promoter.

#### Split protease-based assays

In the EV cargo delivery (EVCD) assay^[75,76,121]^, a split luciferase is used^[120,122]^. A small fragment of NanoLuc (HiBiT) is fused to EV tetraspanins, while the large subunit of NanoLuc (LgBiT) is expressed in recipient cells. When the HiBiT-tagged tetraspanins are exposed to the cytosol of recipient cells, the luciferase fragments combine and emit luminescence signals when in the presence of a substrate. Because NanoLuc produces much brighter luminescence than conventional luciferases such as firefly or Renilla luciferases, NanoLuc-based assays are sensitive enough to detect the rare event of EV cargo delivery.

Perrin *et al.* used an interesting split protease-based method to shed light on the process of back fusion of ILVs in which ILVs were shown to fuse back with the limiting membrane (LM) of the MVB^[87]^. They engineered an NLS-GFP-TCS-CD63 fusion protein consisting of CD63 tagged with GFP containing an N-terminal nuclear localization signal (NLS) and a C-terminal tobacco etch virus (TEV) protease-specific cleavage site (TCS). In cells that co-expressed NLS-GFP-TCS-CD63 and split TEV protease, ILVs were formed that contained NLS-GFP-TCS in their lumen, which upon back fusion, would become exposed to the cytosol. Upon the addition of a protease dimerizer, TEV protease in the cytosol was activated, resulting in the cleavage and release of NLS-GFP from cytosolically exposed NLS-GFP-TCS-CD63. Subsequent nuclear accumulation of NLS-GFP served as a measure for ILV back fusion with the MVB limiting membrane. In this way, the authors showed that ~30% of ILVs underwent back fusion. Interestingly, Joshi *et al.* showed a similar extent of back fusion (~24%) for sEVs internalized by recipient cells^[22]^. How back fusion of ILVs and sEVs is controlled is a central question that remains to be answered, as was discussed above.

Interestingly, it was reported that a maximum of a third of sEVs are generated from dynamic ILVs (i.e., backfused and then regenerated into ILVs) while the majority come from inert ILVs (i.e., that have not undergone back-fusion and new formation in MVBs)^[87]^. Do these dynamic ILVs, after their secretion and subsequent uptake by recipient cells, represent the population of EVs that undergoes back-fusion? Alternatively, there may exist MVBs that permit back-fusion, while other MVBs are non-permissive. Currently, we do not know if inert ILVs and dynamic ILVs arise from the same MVB or distinct MVBs. It is not unimaginable to think that there is a decision-making process in MVBs directing different populations over different trajectories. It is important to find the answers to these questions in order to elucidate the mechanisms behind ILV (re)formation and ILV/EV back-fusion to improve our understanding and the translation of EV-mediated cargo delivery.

### Techniques to study endosomal permeabilization

#### Galectin-based assay

Galectins, including galectin (GAL) 1, GAL3, GAL4, GAL8 and GAL9, are beta-galactoside-binding proteins that are employed as markers for endosomal and lysosomal damage. Because beta-galactosides are exclusively present within the endo/lysosomal lumen, the expression of fluorescent fusion proteins of galectins in the cell cytosol will result in the formation of fluorescent punctae upon galectin accumulation in permeabilized endosomes^[123]^. GAL8 and GAL9 were shown as the more sensitive markers for endosomal permeabilization compared to GAL1, GAL3 and GAL4 for lipoplexes^[123]^, lipid nanoparticles^[123,124]^ and cholesterol-conjugated siRNA^[125]^.

In HEK293T cells genetically engineered to express monomeric azami green-tagged GAL3 (mAG-GAL3), endosomal permeabilization was studied upon incubation with sEVs, but no endosomal permeabilization was detected^[22]^. Immuno-labeling against GAL3 in HeLa cells exposed to sEVs similarly did not reveal GAL3 accumulation in EV-containing endosomes^[59]^. These results suggest that EVs release their cargo from endosomes in a non-destructive manner, which fits with the involvement of back fusion in EV cargo release.

All methods discussed above mainly focus on the direct visualization of membrane fusion or cargo release [Table 2], which is undoubtedly important to understand the underlying compartments and processes in EV cargo release. However, verifying the functionality of these cargoes is indispensable as that is the final outcome of interest. De Jong *et al.* developed the CROSS-FIRE system to detect EV-mediated RNA delivery^[126]^. Using the CROSS-FIRE system, they revealed important molecular determinants of the process of EV-mediated RNA delivery. Rho GTPases Rac1 and RhoA, PAK1, Cav1, ITGB1, Rab5 and Rab7, and ROCK1 were found to be important for functional RNA delivery. Unfortunately, the cellular uptake nor endosomal escape was measured, making it impossible to decide at which step in the delivery process the different proteins play a role.

**Table 2:** Assays to detect membrane fusion, cargo exposure, subcellular site of cargo release, and functional RNA delivery by EVs

**Assay type**		**Mechanism**	**Advantages**	**Limitations**	**Ref**
Fluorescence quenching based on R18	Membrane fusion	Lipid-mixing/ Fusion of the PS:PC:Chol-R18 liposomes with lipid membranes restores fluorescence	Simple and cost-effective	Does not address cellular location of cargo release	^[58,117]^
DiI-DiD-based FRET assay	Membrane fusion (EVs with LUVs)	Lipid-mixing/DiI and DiD as FRET pair (donor intensity increases upon fusion)	Simple and cost-effective; Can be combined with Cryo-TEM to investigate fusion mechanism	Does not address cellular location of cargo release	^[68]^
Nanobody assay; CLEM	EV cargo exposure; detection of subcellular site of cargo release	Punctate structure formation upon binding of anti-GFP fluobody (expressed in recipient cells) to GFP cargo from GFP-CD63 EVs	Simple analysis of cargo exposure; Direct evidence of cargo release; Pinpoints subcellular site of EVs cargo release by means of CLEM	Time-consuming in the case of combination with CLEM	^[22]^
Retrofusion assay	Membrane fusion (ILV retrofusion)	Nuclear accumulation of NLS-GFP in NLS-GFP-TCS-CD63-expressing cells that co-express split TEV protease, upon addition of protease dimerizer	Measures ILV retrofusion	Complex; Nuclear GFP signal derives from PM, MVB and retrofused ILV membranes; many controls needed; Requires live cell imaging and thresholding and normalization for analysis	^[87]^
Galectin assay	Endosomal permeabilization	Endosomal accumulation of mAG-galectin in mAG-galectin expressing cells upon endosomal permeabilization, i.e., exposure of cytosolic galectins to β-galactosides present exclusively in the endo/lysosomal lumen.	Pinpointing site of endosomal permeabilization/cargo release	Small endosomal perforations may remain undetected	^[22]^
Cre-recombinase	mRNA delivery	RFP to GFP fluorescence conversion in recipient reporter cells upon addition of Cre-recombinase mRNA-containing EVs	Detects functional delivery	EVs can be contaminated with Cre-recombinase protein	^[127,128]^
CROSS-FIRE	sgRNA delivery	mCherry to GFP fluorescence conversion in recipient reporter cells upon addition of sgRNA-containing EVs	Detects functional delivery; no contamination of EVs with protein	More complex genetic engineering of producer and recipient cells; CRISPR/cas-based assay	^[126]^
REMD	mRNA delivery	Luciferase expression in recipient reporter cells upon delivery of EVs containing NanoLuc mRNA with a stop codon	Detects functional delivery; no contamination of EVs with protein	More complex genetic engineering of producer and recipient cells; CRISPR/Cas-based assay; mRNA is relatively large and not the most important natural cargo of EVs	^[75,76,121]^

LUVs: large unilamellar vesicles; TEM: transmission electron microscopy; CLEM: correlative light and electron microscopy; mAG: monomeric azami-green; CROSS-FIRE: CRISPR operated stoplight system for functional intercellular RNA exchange; REMD: RNA-editing-based mRNA delivery.

### Read out systems for functional delivery of RNA cargo

#### RNA editing-based assays

Using an inventive assay in which cancer cells were engineered to secrete Cre-recombinase mRNA-containing EVs to induce Cre-induced RFP to GFP fluorescence conversion in recipient reporter cells, Zomer *et al.* showed *in vivo* cell-cell communication through EVs^[127,128]^. However, translation of the mRNA in EV producer cells may result in co-loading of the mRNA-encoded protein in EVs^[129]^. To circumvent the need for mRNA translation in the readout of functional RNA delivery and prevent the possible contamination with mRNA-encoded protein in EVs, De Jong *et al* developed a CRISPR/Cas9-based strategy which they termed CROSS-FIRE (CRISPR-O>perated Stoplight System For Functional Intercellular RNA Exchange)^[126]^. In this method, EVs were engineered to contain (non-coding) single guide RNA (sgRNA) that, upon delivery into Cas9-expressing recipient cells, caused mCherry to GFP fluorescence conversion by virtue of CRISPR/Cas9-directed frameshifting. The experiments included a 10-day co-culture of sgRNA-EV producer cells and Cas9-reporter recipient cells, and direct addition of donor EVs to reporter cells every 72 h for 12 additions with an average dose of 1.1e11 ± 4.9e10 EVs, to facilitate the detection of changes in GFP fluorescence.

Another RNA-editing-based assay was recently developed to investigate EV-mediated mRNA delivery into recipient cells, the REMD assay^[75,76,121]^. EV producer cells were transfected with a Nanoluciferase gene containing a stop codon, preventing luciferase expression. Recipient cells were made to express CRISPR/Cas13 and sgRNA targeting the mutated Nluc mRNA. In the case of successful cargo delivery by EVs, the mutated Nluc mRNA was converted into translationally active mRNA by the presence of CRISPR/Cas13 and sgRNA in the recipient cells, resulting in luciferase expression. In this way, the contamination of EVs with luciferase protein (as may occur when EV producer cells overexpress non-mutated Nluc mRNA) was effectively prevented, and luciferase expression in recipient cells rightfully reflected EV-mediated mRNA delivery.

Of note, the above-mentioned RNA editing-based assays are useful for quantifying EV-mediated RNA delivery [Table 2], but are not capable of pinpointing the subcellular sites of cargo delivery. Therefore, a combination of techniques is needed to correlate EV uptake, intracellular trafficking and escape of EV cargo from endosomes with the induction of phenotypic changes in recipient cells, i.e., functional delivery of EV cargo.

### Controls in cargo delivery assays

For functional cargo delivery assays, it would be helpful to include a dose curve to determine if an increase in the functional outcome correlates with an increase in the added amount of EVs. The amount of EVs should be based not only on the total protein content but also on the number of particles, e.g., measured by Nanoparticle Tracking Analysis (NTA), because contaminants can lead to an overestimation of the EV protein content^[130]^. EVs should be used only after thorough characterization following MISEV(2018) guidelines to enable the reproducibility of experiments among different labs. Briefly, markers typically enriched in EVs (CD9, CD81, TSG101, ALIX) and the absence of markers for specific organelles (endoplasmic reticulum, Golgi) should be demonstrated. A morphological investigation should be performed with electron microscopy. Further EV purification after ultracentrifugation should be done with sophisticated techniques such as sucrose gradient centrifugation, ultrafiltration, or affinity chromatography. The choice of a specific technique depends on the cargo being loaded in the EVs. For miRNA, siRNA, or mRNAs, it is indispensable to remove protein-nucleic acid aggregates that may co-precipitate with EVs in ultracentrifugation and can be separated, e.g., with sucrose gradient centrifugation.

To demonstrate the presence of EV cargo in its lumen and to eliminate the possibility of its association on the EV surface, controls such as nuclease- (DNase, RNase), proteinase K-, and lipase-treated EVs, depending on the cargo under investigation, should be included. When studying the natural mechanisms of EV-mediated cell-cell communication, a physiologically relevant amount of EVs must be used, as excessive amounts of EVs may give rise to artifacts and non-physiological outcomes, including toxicity. This may also apply to EVs being used as drug carriers. It is still a matter of debate whether the 10 μg of EVs (equivalent to 10^11^ particles) used in many in vitro experiments is a physiologically relevant amount. The yield of EVs from raw materials (culture supernatant or body fluid) is often quite low (0.5 μg of protein/mL of supernatant), and thus extensive enrichment of EVs is typically performed^[88]^. Also, multiple producer-recipient cell combinations could be tested to show the generality of the method as well as the mechanism being put forward.

It is of utmost importance to analyze the stoichiometry of the whole process: number of EVs added, number of cargo molecules per EV, % of uptake, % of EVs that release cargo, the (approximate) number of cargo molecules released, functional effect, etc. This would greatly help to reach a comprehensive understanding of EV-mediated cargo delivery. For some cargoes, it is relatively easy to obtain these data, for example, for mRNAs (by RT-qPCR), while it is more challenging for others, such as for miRNA due to, e.g., possible interference from endogenous miRNAs.

The investigation of EV-mediated mRNA delivery may suffer from undesired co-loading of translated protein into EVs. Also, mRNA-protein aggregates can form and co-precipitate with EVs and overestimate the outcomes. This can give rise to misleading results by giving false positives. To confirm that the outcome is from mRNA and not from proteins, EVs loaded with mRNAs can be added to recipient cells transfected with siRNA against the reporter mRNA. If the protein quantity is reduced in these conditions, it is a good indication that the outcome results from mRNA and not the protein. Similarly, cycloheximide, which stops the de novo synthesis of proteins, can also be used in recipient cells to analyze the contribution of newly synthesized proteins resulting from mRNA delivery. Another way to investigate the functional delivery of mRNA rather than protein is to treat EVs with heat shock and methylene blue^[117,131,132]^ which penetrates the lumen and interferes with the RNA. Additionally, EVs can be treated with propidium monoazide (PMA)^[117,133]^, which is an impermeant nucleic acid intercalating photoreactive dye that blocks RT-qPCR. If it affects mRNA amplification, this indicates that the mRNA is present on the surface of EVs rather than in the lumen. For miRNAs, nonendogenous miRNAs (viral miRNAs) may be considered as cargo to allow separation of its effects from endogenous miRNAs^[119]^. To quantify EV RNA delivery, one can radioactively label EV RNA by incubation of EV producer cells with 3H-labeled uracil^[134]^.

## COMPARISON OF EVS WITH OTHER NANOPARTICLES

Do EVs share similarities with other nanoparticles, such as viral particles and synthetic gene delivery vectors, in their uptake and cargo release dynamics? In an interesting study, Murphy *et al.* tackled this question by comparing an FDA-approved cutting-edge lipid nanoparticle (LNP) formulation with EVs in terms of uptake and cargo (specifically gRNA) delivery^[135]^. With this study, they addressed the issue that EVs are known to contain very low amounts of miRNA, i.e., only 1 copy per hundreds or thousands of EVs^[135,136]^, making the scientific community question whether EVs can bring about a real change in cell phenotype, despite several reports showing EV-mediated changes in the phenotype of recipient cells^[1-3]^, hinting at high efficiency of cargo release by EVs. Indeed, Murphy *et al.* showed that when the particle-to-gRNA ratio is kept the same, EVs are more efficient in imparting a functional effect, indicating successful cargo delivery, than LNPs. In fact, LNPs containing less than 1 pM gRNA did not result in any functional effect, while EVs containing 0.1-2.5 fM gRNA did. The authors concluded that EV-mediated gRNA delivery was at least 2-fold more efficient than delivery mediated by LNPs. Put the other way around, the development of a synthetic gene delivery vector, i.e., LNPs, with an endosomal escape capacity almost equal to that of a natural delivery vector, i.e., EVs, can be considered a big achievement.

Studies suggested that EVs or EV-like liposomes are more efficiently taken up by cells than LNPs^[137,138]^. About 10% of EVs were taken up in 2 h and 26%-32% in 24 h; in contrast, 0.6% of LNPs were taken up in 2 h and 1% in 24 h. Thus, it appears that the uptake, as well as the efficiency, of cargo delivery of EVs, is generally higher than that of artificial delivery vehicles such as LNPs. About only 1%-2% of endocytosed LNPs undergo endosomal escape to release their cargo^[139]^, and this is the rate-limiting step for their efficacy. EVs similarly use endocytosis for uptake and cargo release efficiency turns out to be ~25%^[22]^, an order of magnitude higher than with LNPs.

An important point for consideration is the amount of EVs that is added to recipient cells in order to induce a phenotypic effect. For example, multiple doses of EVs were required in the CROSS-FIRE system to bring about the shift from RFP to GFP expression^[126,140]^. And even then, only a small percentage of cells showed reporter gene (GFP) expression. Do these large amounts of EVs represent the physiological situation and is this question even relevant? We believe the question is relevant if the physiological roles of EVs, e.g., their role in metastasis^[52]^, are under investigation, but may be less relevant when EVs are exploited for drug delivery purposes, provided that the large amounts of EVs do not induce toxic side effects.

Overall, the downside of using assays that depend on the downstream effects of the cargo is the fact that the measured functional effect does not necessarily reflect the efficiency of cargo delivery, but may be influenced by other factors. This can be illustrated by an example from the gene delivery field: endosomal escape of pDNA into the cytosol does not necessarily result in the expression of the DNA if it is not successfully transferred to the cell nucleus, for transcription to take place. Therefore, the transfection efficiency does not necessarily reflect the efficiency of DNA delivery into the cytosol. Likewise, high cellular uptake of gene delivery vectors does not necessarily correlate with high transfection efficiency, because the endosomal escape of the genetic cargo also needs to be efficient^[109]^. Therefore, it is important to analyze the cellular uptake of nanoparticles, the intracellular release of the cargo, and the instigation of a functional effect altogether. The combined quantitative assessment of the different steps in EV cargo trafficking and functional effects is key in the elucidation of the spatiotemporal dynamics of EVs in cells^[118]^.

Various factors can impact the EV uptake and cargo delivery process. Upstream (donor/producer cell type, cargo expression and loading efficiency, isolation procedure, *etc*.) as well as downstream factors (acceptor/recipient cell type, microenvironment, *etc*.) play a role in deciding the fate of EVs in recipient cells. Thus, there is a growing need for a golden standard assay to analyze and quantify EV-mediated cargo delivery and its functional outcome. Ideally, such an assay should be simple, sensitive, quantitative, and scalable.

## SUMMARY AND OUTLOOK

EVs display a natural homing/targeting capacity together with the potential to deliver cargo molecules into cells, thereby providing a unique platform for the delivery of therapeutic agents to even challenging-to-reach organs like the brain^[141]^. However, the composition of EVs is heterogeneous, which can impact their overall functionality as well as trigger undesired side effects. Additionally, although progress has been made, a significant obstacle to clinical applications of EVs is the challenge of obtaining high quantities of EVs at a reasonable cost and with consistency between batches. The development of universal or patient-matched EV donor cell lines could be helpful, considering the presence of Human Leukocyte Antigens (HLAs) on EVs^[142]^. A thorough and standardized isolation and characterization procedure is essential to the development of EVs as drug delivery systems a.o. to reveal the EV population that is responsible for exerting functional effects. In addition, further understanding of the mechanisms of EV biogenesis, cellular internalization and intracellular fate is needed. To obtain a comprehensive understanding of EV-mediated cargo release, it is advisable to examine relevant producer-recipient cell combinations and analyze the stoichiometry of the delivery process. Most importantly, the delivery process needs to be studied under conditions that mimic the in vivo situation and/or be verified in vivo, which is not common practice^[143]^. As an example, the influence of bodily fluids (blood, plasma, CSF, *etc*.) needs to be reflected in the experiments, which will certainly add another layer of complexity. While exosomes are naturally present in saliva, urine, blood, and plasma, in the majority of experiments, their mechanism of action is investigated under standard cell culture conditions in physiological buffers, neglecting the influence of environmental factors present in their natural extracellular surrounding. This includes the presence of specific macromolecules in the extracellular fluid. For synthetic nanoparticles, it is well-known that a protein corona is formed upon administration of the nanoparticles in biological fluids, e.g., blood plasma, which is known to influence the biodistribution of these nanoparticles^[144-146]^. Recently, the formation of a protein corona on EVs in blood plasma was shown^[147]^. As the nanoparticle protein corona composition is, amongst others, influenced by nanoparticle size and surface chemistry, it is easy to envision that the investigation of EV protein corona constitutes a new challenge to the field, forming an emerging subject area for EV research^[148]^.

## DECLARATIONS

### Copyright

© The Author(s) 2023.
