# Erratum: Breaking free: endocytosis and endosomal escape of extracellular vesicles

**DOI:** 10.20517/evcna.2023.42

**Published:** 2023-09-12

**Authors:** Laís Ribovski, Bhagyashree Joshi, Jie Gao, Inge Zuhorn

**Affiliations:** ^1^Department of Biomedical Engineering, University Medical Center Groningen, University of Groningen, Groningen 9713 AV, the Netherlands.; ^2^Department of Bionanoscience, Kavli Institute of Nanoscience, Delft University of Technology, Delft 2629 HZ, the Netherlands.; ^#^Authors contributed equally.



The authors want to make the following corrections to this paper^[1]^.

In the section “Intracellular trafficking of EVs”, the citations after the sentence “Using another cell-free assay, Morandi *et al.* provided interesting insights into the process of endosomal fusion in EV cargo release”and “Murphy *et al.* tackled this question by comparing an FDA-approved cutting-edge lipid nanoparticle (LNP) formulation with EVs in terms of uptake and cargo (specifically gRNA) delivery” were missed.

Correctly modify as follows:

Using another cell-free assay, Morandi *et al.* provided interesting insights into the process of endosomal fusion in EV cargo release^[22,68,87]^.

Murphy *et al.* tackled this question by comparing an FDA-approved cutting-edge lipid nanoparticle (LNP) formulation with EVs in terms of uptake and cargo (specifically gRNA) delivery^[135]^. Corresponding subsequent citation numbers also need to be adjusted: reference 140 is changed to reference 135, reference 135 is changed to reference 136, reference 136 is changed to reference 137, reference 137 is changed to reference 138, reference 138 is changed to reference 139, and reference 139 is changed to reference 140.

We apologize for any inconvenience caused and state that the scientific conclusions are unaffected. The original article has been updated.
